# Supplementary material for: CLPB3 is required for the removal of chloroplast protein aggregates and thermotolerance in Chlamydomonas
Source: J Exp Bot. 2023 Mar 23;74(12):3714–28. doi: 10.1093/jxb/erad109 (PMC10299786; doi:10.1093/jxb/erad109)
Supplement: erad109_suppl_Supplementary_Table_S1_Figures_S1-S6 [file erad109_suppl_supplementary_table_s1_figures_s1-s6.pdf]

## SUPPLEMENTARY INFORMATION

**Title:** CLPB3 is required for the removal of chloroplast protein aggregates and for thermotolerance in *Chlamydomonas*

**Authors:** Elena Kreis[1], Justus Niemeyer[1], Marco Merz[1], David Scheuring[2], Michael Schroda[1, ¶]

**Affiliations:**

[1] Molekulare Biotechnologie & Systembiologie, TU Kaiserslautern, Paul-Ehrlich Straße 23, D-67663 Kaiserslautern, Germany

[2] Phytopathologie, TU Kaiserslautern, Paul-Ehrlich Straße 22, D-67663 Kaiserslautern, Germany

[¶] Corresponding author: Michael Schroda (schroda@bio.uni-kl.de)

**Supplementary Table S1.** Primers used for cloning and genotyping. Lower case letters indicate nucleotides differing from the template.

| Primer # | Primer name | Nucleotide sequence (5'→3') |
|---|---|---|
| #1631 | CLPB3-Eco | gaaagaattCGCgGCGCAGTCCGGCTCCGGCGGCGGC |
| #1632 | CLPB3-Hind | GCGCaagCTTTGCTCCGCTCCCTCGACCTATGC |
| #1466 | Control locus_for | ATGCTTCTCTGCATCCGTCT |
| #1467 | Control locus_rev | ATGTTTTACGTCCAGTCCGC |
| #1600 | CLPB3_F5 | TGTGAATGGCAACGACGGTA |
| #1604 | CLPB3_R4 | TGCCCGTGTCTAAATTGCCT |
| #1462 | OMJ 913 | GCACCAATCATGTCAAGCCT |
| #1463 | OMJ 944 | GACGTTACAGCACACCCTTG |
| #1819 | CLPB3-2_For | CTTAGGGAGTGGCGTAGCTG |
| #1820 | CLPB3-2_Rev | CCTGCACATGCATACCAAAC |
| #1462 | OMJ 913 | GCACCAATCATGTCAAGCCT |
| #1463 | OMJ 944 | GACGTTACAGCACACCCTTG |
| #1613 | CIB1for | AGGCTTGACATGATTGGTGC |
| #1614 | CIB1rev | CAAGGGTGTGCTGTAACGTC |
| #1615 | CIB1in_rev | TCCAGATCCTCCAGGTCAAC |
| #1616 | CIB1in_for | GTGAGCAACGTCTGGATGTC |

## SUPPLEMENTARY FIGURES AND FIGURE LEGENDS

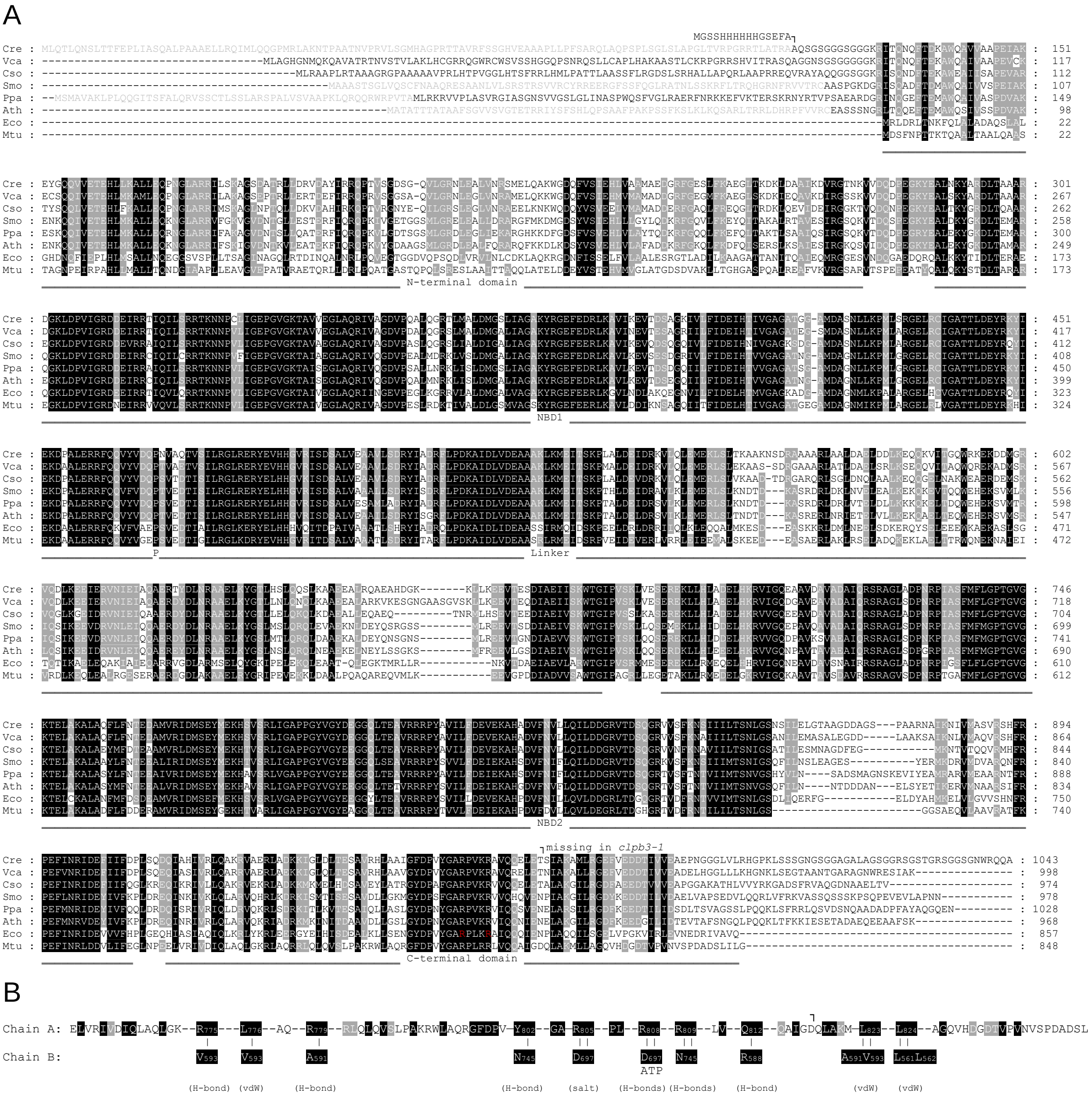


**Supplementary Fig. S1.** (A) Alignment of amino acid sequences of chloroplast and bacterial ClpB proteins. Residues highlighted in black and gray are conserved in eight and six of the sequences, respectively. Chloroplast transit peptides predicted by TargetP are shown in gray letters. The sequence with a hexahistidine tag, replacing the predicted chloroplast transit peptide of *Chlamydomonas* CLPB3, was used for the production of recombinant CLPB3 in *E. coli*. The regions in *E. coli* ClpB comprising N- and C-terminal domains, nucleotide binding domains NBD1 and NBD2, and the linker are indicated. C-terminal sequences missing in the *clpb3-1* mutant are marked. Truncation of the C-terminal domain in *E. coli* ClpB abolished hexamer formation, while hexamer formation was impaired by mutation of the arginines shown in red letters to alanines (Barnett *et al.*, 2000; Barnett and Zolkiewski, 2002). Ath – *Arabidopsis thaliana* (At5g15450), Cre – *Chlamydomonas reinhardtii* (Cre02.g090850), Cso – *Chlorella sorokiniana* (PRW59672), Eco – *Escherichia coli* (P63284), Mtu – *Mycobacterium tuberculosis* (WP_003401905), Ppa – *Physcomitrium patens* (XP_024363741), Smo – *Selaginella moellendorfffii* (XP_002988985), Vca – Volvox carteri (Vocar.0001s1202). Alignments were done using CLUSTAL W and displayed by GeneDoc. (B) Interactions between residues of the C-terminal domain of a ClpB protomer (chain A) with its neighboring protomer (chain B) according to the cryo-EM structure (PDB 6W6G) of *Mycobacterium tuberculosis* ClpB (Yin *et al.*, 2022). vdW – van der Waals interactions.

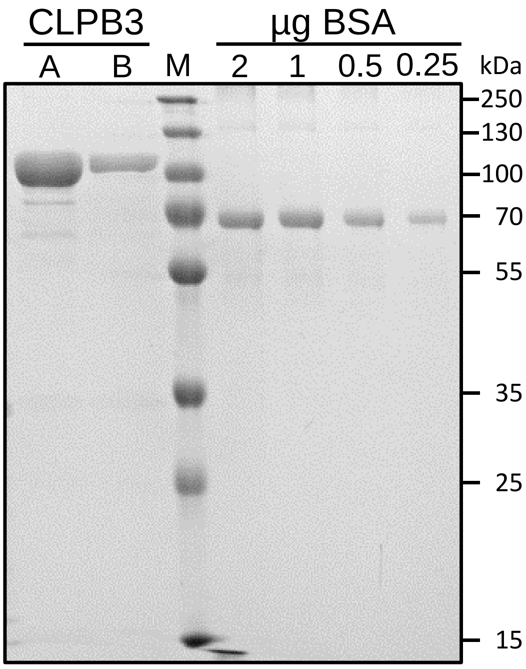


**Supplementary Fig. S2.** Production of recombinant CLPB3 in *E. coli. Chlamydomonas* CLPB3 was expressed with a N-terminal hexa-histidine tag in *E. coli* and purified by cobalt-nitrilotriacetic acid affinity chromatography followed by gel filtration on an Enrich SEC650 column. 2 µl of proteins resulting from two independent preparations (A and B) were analyzed next to a dilution series of BSA on a 12% SDS-polyacrylamide gel and stained with Coomassie blue.

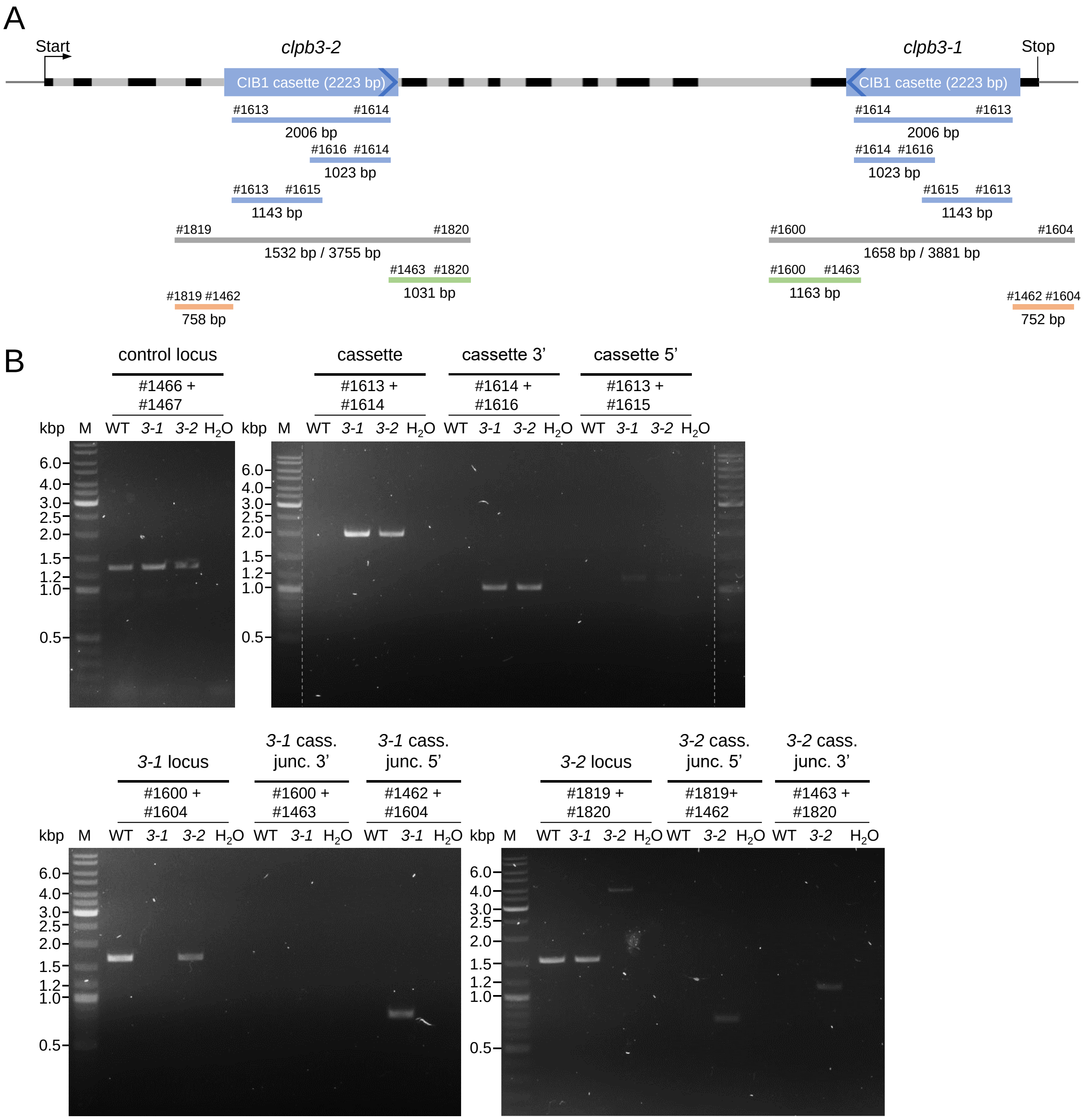


**Supplementary Fig. S3.** Analysis of the CIB1 integration sites in the *CLPB3* gene of mutants *clpb3-1* and *clpb3-2* by PCR. (A) Gene model of the *CLPB3* gene with exons shown as black boxes and introns as grey boxes. The integration site of the CIB1 cassette in the fourth intron (*clpb3-2*) and in the 12$^{th}$ exon (*clpb3-1*) are shown. Bars indicate the expected PCR products from the CIB1 cassette (blue), wild-type DNA (grey), the 5' junction (orange), and the 3' junction (green). Numbers on top of the bars indicate the expected sizes of PCR products and the primer number as provided in Table S1. (B) PCR products on genomic DNA from wild type (WT) and the *clpb3* mutants.

```
AACCCCCCCGAAAGCATGCAAGCTACCTGTTGCAACCAATGTCTTTGCGTCACATTAGCGAG

           HSE2                                            HSE1
TTCGGGTACGTTCTGGCACGCGTGGCCTGGACTTATCGCGTGGATTCTGGACTCCCGTGAGG
GCCACTCGCAAAACCTTTCAACCCCGGCGCGGGTATATATAAAGATATGTATTCCTCTACGC
CATCCAATTAATTACGCACACCGCAAACGCTTATGCTTCAGACGCTTCAAAACAGCCTAACA
ACTTTTGAGCCGCTTATCGCTTCCCAGGCCCTGCCAGCAGCAGCCGAACTCCTTCGCCAAAT
CATGCTACAGCAAGGT-Intron 1-AG
```

**Supplementary Fig. S4.** Putative heat shock elements (HSEs) in the *CLPB3* promoter. Sequences contributing to the canonical nGAAn / nTTCn repeats in HSEs are shown in red. Putative HSEs are underlined. The putative TATA-box is shown in underlined bold letters. The 5'UTR is in green letters and the coding region is highlighted in green. The position of the first intron is shown.

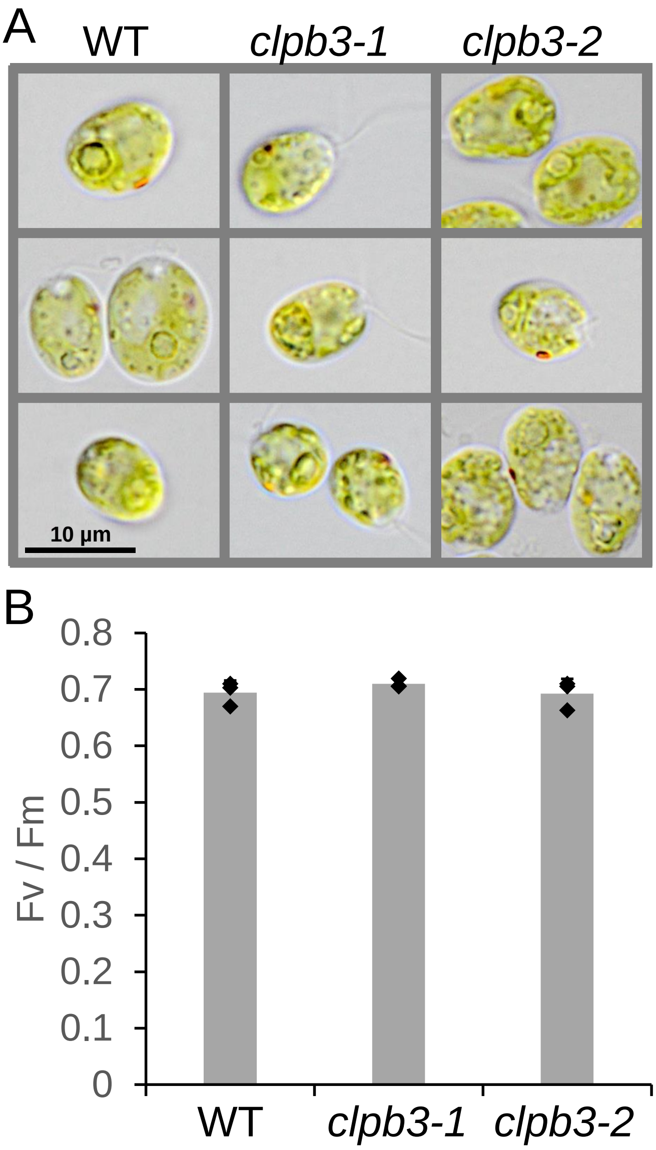


**Supplementary Fig. S5.** *Chlamydomonas clpb3* mutants display no obvious phenotype regarding chloroplast development and PSII activity. (A Light microscopy images of cells grown in TAP medium under ambient conditions to a density of ~8 x $10^6$ cells $mL^{-1}$. (B) Comparison of Fv/Fm values. Shown are averages from three independent experiments, error bars indicate SD. There were no significant differences (T-test, $p > 0.05$).

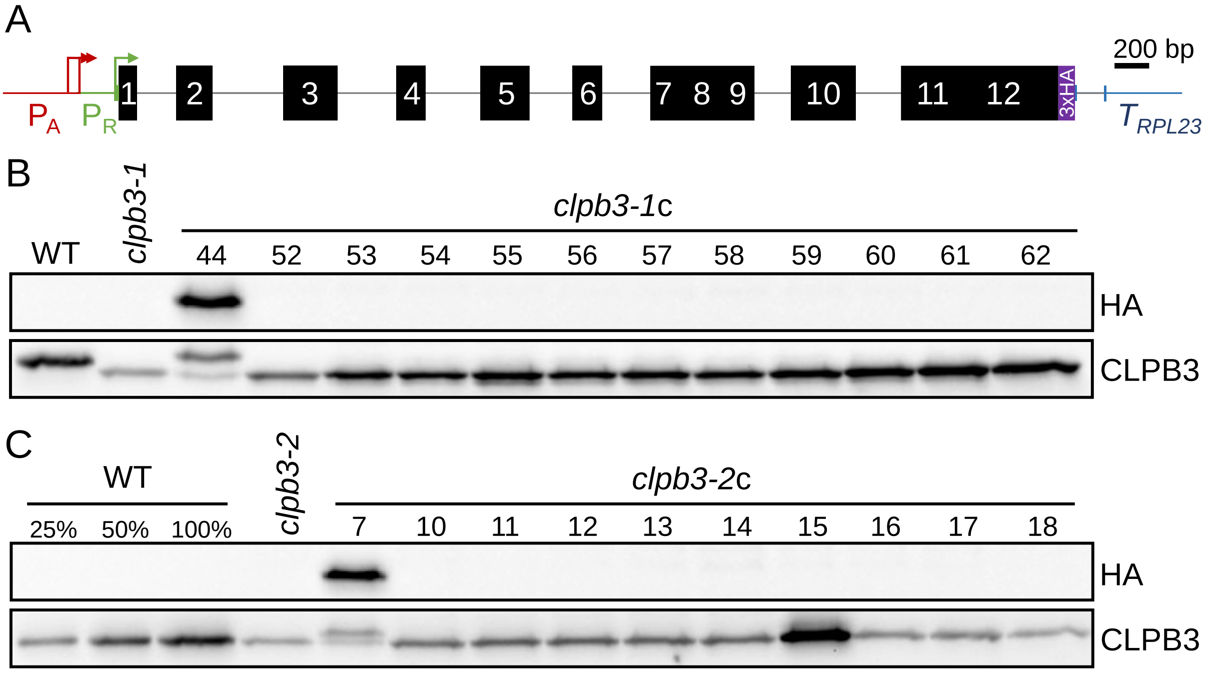


**Supplementary Fig. S6.** Screening for complemented *clpb3* mutant lines. (A) Construct used for complementation. For details, see Fig. 2A. (B) Immunoblot analysis of transformants generated with the construct shown in (A). Whole-cell proteins of wild type (WT), *clpb3-1* mutant, and 12 randomly picked transformants were separated on a 12% SDS-polyacrylamide gel and analyzed by immunoblotting using antibodies against the HA epitope and CLPB3. (C) Same as in (B), but with ten randomly picked *clpb3-2* mutants transformed with the construct shown in (A).
